# Postmenopausal Osteoporosis: From Molecular Pathways to Therapeutic Targets—A Mechanism-to-Practice Framework Integrating Pharmacotherapy, Fall Prevention, and Adherence into Patient-Centered Care

**DOI:** 10.3390/jcm15010102

**Published:** 2025-12-23

**Authors:** Graziella Ena, Muhammad Soyfoo

**Affiliations:** 1 Department of Gynecology, Hôpital Delta, 1160 Brussels, Belgium; graziellaena@hotmail.com; 2 Department of Rheumatology, Hôpital Universitaire de Bruxelles (HUB), Université libre de Bruxelles (ULB), 1070 Brussels, Belgium

**Keywords:** postmenopausal osteoporosis, pathophysiology, bone mineral density, treatment algorithm, osteonecrosis of the jaw

## Abstract

The next frontier in postmenopausal osteoporosis management lies not in novel pharmacological agents, but in the systematic integration of mechanism-guided drug selection, fall prevention, and long-term adherence strategies into a unified patient-centered care model. This review is intended for clinicians and clinical researchers involved in the diagnosis, treatment, and long-term management of postmenopausal osteoporosis. We provide a mechanism-to-practice framework that explicitly maps each therapeutic class to the specific molecular pathway it targets: bisphosphonates inhibit osteoclast function downstream of RANKL activation; denosumab blocks RANKL directly at the cytokine level; romosozumab inhibits sclerostin to restore Wnt-mediated bone formation. This mechanistic foundation supports a risk-stratified treatment paradigm in which antiresorptives address accelerated remodeling in moderate-risk patients, while patients at very high fracture risk—characterized by severe bone deficit or recent fragility fractures—benefit from an anabolic-first approach followed by consolidation. Beyond drug selection, we examine the persistent treatment gap in which fewer than 20% of post-fracture patients receive therapy, arguing that fall prevention—responsible for >90% of hip fractures—and medication adherence deserve equal priority in clinical practice. We further analyze key controversies, including T-score- versus FRAX-based intervention thresholds, limitations of the trabecular bone score, cost-effectiveness constraints on anabolic-first sequencing, and evidence gaps in post-denosumab transition strategies. By synthesizing mechanistic insights, guideline recommendations, and critical appraisal of current limitations, this review offers not only an overview of existing knowledge but a coherent decision-support model aimed at improving fracture prevention through comprehensive, individualized care.

## 1. Introduction

Postmenopausal osteoporosis constitutes a major global public health concern, characterized by progressive deterioration of bone mass, microarchitectural disruption of skeletal tissue, and consequent increased susceptibility to fragility fractures. The World Health Organization defines osteoporosis based on bone mineral density measurements, with diagnostic thresholds established at 2.5 standard deviations below the young adult mean. However, the clinical significance of this condition extends far beyond densitometric criteria, encompassing the substantial morbidity, mortality, and socioeconomic burden associated with osteoporotic fractures [1,2].

The menopausal transition triggers accelerated bone resorption exceeding formation capacity. During the first 5–10 years following menopause, women experience annual bone loss of 2–3%, substantially exceeding the 0.5–1% annual loss in age-related osteoporosis. This accelerated phase preferentially affects trabecular bone, explaining the earlier occurrence of vertebral fractures relative to hip fractures [3].

The epidemiological burden of osteoporotic fractures is substantial and growing with population aging. Approximately 9 million osteoporotic fractures occur annually worldwide. Hip fractures carry particularly grave consequences, with 1-year mortality of 20–25% and substantial permanent disability. Vertebral fractures, even when clinically silent, predict future fractures and cause progressive kyphosis with pulmonary restriction and chronic pain. Direct costs exceed €37 billion annually in Europe [4,5]. An emerging epidemiological consideration is the relationship between prior joint arthroplasty and subsequent osteoporotic fracture patterns. A recent population-based analysis of hip fracture incidence in Romania (2008–2019) demonstrated that increasing total hip arthroplasty (THA) prevalence modifies observed fracture epidemiology: because the operated hip no longer presents a risk of native hip fracture, rising THA rates create a ‘competing event’ that reduces the pool of hips at risk, thereby affecting age-standardized hip fracture incidence calculations [6]. This phenomenon—whereby the operated hip is protected from osteoporotic fracture while periprosthetic fracture risk emerges as a distinct entity—complicates traditional fracture epidemiology and FRAX-based risk assessment in post-arthroplasty populations. Studies examining periprosthetic bone changes following arthroplasty have identified both protective factors (restored mobility, reduced fall fear) and risk-enhancing factors (periprosthetic bone loss, stress shielding) that necessitate individualized osteoporosis management strategies in this growing patient population. These findings underscore the complexity of fracture epidemiology in contemporary aging populations.

Despite effective diagnostic tools and therapies, osteoporosis remains substantially underdiagnosed and undertreated. Fewer than 20% of high-risk patients receive appropriate therapy following fragility fracture. This gap reflects inadequate screening, underrecognition of fracture significance, concerns about rare complications, and fragmented care [7]. This review advances a central thesis: the next frontier in postmenopausal osteoporosis management is not newer drugs, but the integration of mechanism-guided pharmacotherapy, fall prevention, and medication adherence into a unified, patient-centered care model. We provide a mechanism-to-practice framework explicitly connecting the three molecular pathways activated by estrogen deficiency—RANKL/OPG dysregulation, inflammatory cytokine cascades, and sclerostin-mediated Wnt inhibition—to their corresponding therapeutic targets. Critically, we contend that fall prevention deserves equal emphasis to drug selection: over 90% of hip fractures result from falls, yet fall risk assessment and intervention remain marginalized in most osteoporosis care pathways. Throughout, we adopt a critical stance on persistent controversies—T-score versus FRAX-based thresholds, the cost-effectiveness of anabolic-first strategies, evidence gaps in sequential therapy—rather than merely cataloging guideline recommendations. The goal is to equip clinicians with an integrated framework for individualized care, not simply a summary of existing knowledge.

## 2. Pathophysiology of Postmenopausal Bone Loss

### 2.1. Estrogen Deficiency and the RANKL-OPG Axis

Estrogen exerts profound regulatory effects on bone metabolism through direct actions on all cellular components of the bone remodeling unit. Estrogen receptors, both alpha and beta subtypes, are expressed throughout the skeleton on osteoblasts, osteoclasts, and osteocytes, mediating effects on cellular survival, differentiation, and functional activity. The withdrawal of estrogenic influence following menopause fundamentally disrupts the homeostatic balance between bone formation and resorption that maintains skeletal integrity throughout adult life [8].

**Figure 1:**
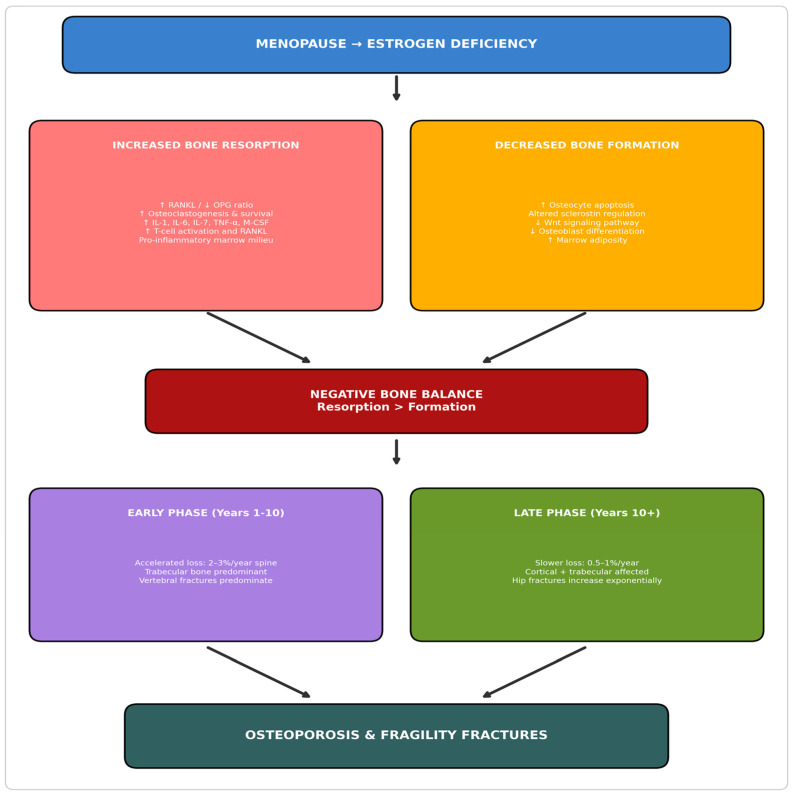
Pathophysiology of Postmenopausal Osteoporosis. This figure illustrates the pathophysiological cascade from menopause-induced estrogen deficiency to osteoporosis and fragility fractures. Arrows indicate the direction of pathophysiological progression and causal relationships between processes. Color coding: Blue represents the initiating event (estrogen deficiency); Red indicates processes of increased bone resorption; Orange indicates decreased bone formation; Dark red represents the resulting negative bone balance; Purple and olive green distinguish the early (trabecular-predominant) and late (cortical involvement) phases of bone loss, respectively; Dark slate represents the final clinical outcome. Abbreviations: IL, interleukin; M-CSF, macrophage colony-stimulating factor; OPG, osteoprotegerin; RANKL, receptor activator of nuclear factor kappa-B ligand; TNF-α, tumor necrosis factor-alpha.

The primary molecular consequence of estrogen deficiency is enhanced osteoclastogenesis through alterations in the RANKL-OPG signaling axis. Under physiological conditions, estrogen suppresses RANKL (receptor activator of nuclear factor kappa-B ligand) production by osteoblastic lineage cells, bone marrow stromal cells, and T-lymphocytes while simultaneously stimulating osteoprotegerin (OPG) secretion. OPG functions as a decoy receptor that binds RANKL and prevents its interaction with RANK on osteoclast precursors. The resulting shift in the RANKL-to-OPG ratio following menopause favors osteoclast precursor recruitment, differentiation, activation, and prolonged survival, leading to enhanced bone resorption at each remodeling site [9] (Figure 1).

### 2.2. Inflammatory Cytokine Activation

Estrogen deficiency creates a pro-inflammatory state within the bone marrow microenvironment that amplifies osteoclastic bone resorption. Production of pro-inflammatory cytokines including interleukin-1 (IL-1), interleukin-6 (IL-6), interleukin-7 (IL-7), tumor necrosis factor-alpha (TNF-α), and macrophage colony-stimulating factor (M-CSF) increases substantially following estrogen withdrawal. These cytokines promote osteoclastogenesis through both RANKL-dependent and RANKL-independent pathways. TNF-α, in particular, synergizes with RANKL to enhance osteoclast differentiation and directly promotes osteoclast survival [10].

T-lymphocytes play an increasingly recognized role in estrogen deficiency-induced bone loss. In the estrogen-deficient state, T-cells become activated and represent significant sources of TNF-α and RANKL within the bone marrow. Estrogen normally suppresses T-cell activation and cytokine production through direct effects on T-cell estrogen receptors and indirect effects through antigen-presenting cells. The loss of this immunomodulatory function establishes a self-amplifying inflammatory cycle that sustains elevated bone resorption beyond the initial estrogen withdrawal period [11].

### 2.3. Osteocyte Dysfunction and Sclerostin

Osteocytes comprise >90% of bone cells and orchestrate bone remodeling through mechanosensory capabilities and signaling molecule production. Embedded within mineralized matrix in an extensive lacunocanalicular network, they sense mechanical strain and coordinate bone formation and resorption. Estrogen withdrawal accelerates osteocyte apoptosis, disrupting this network and impairing skeletal adaptation to mechanical demands [11,12].

**Figure 2:**
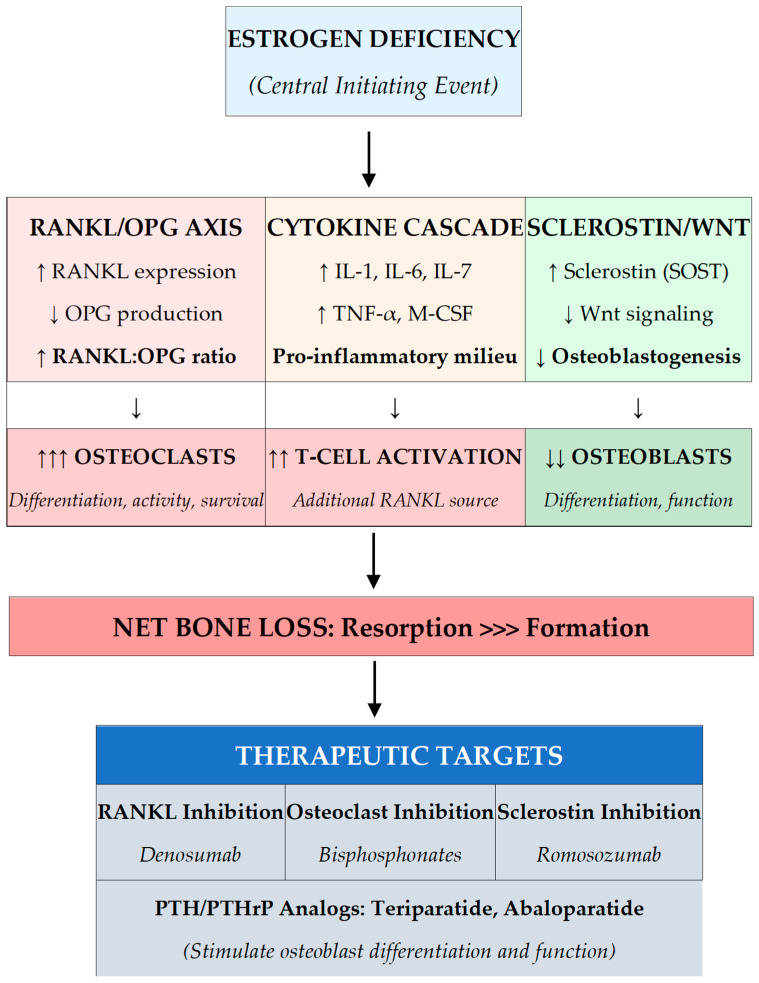
Molecular Pathways in Postmenopausal Osteoporosis: From Estrogen Deficiency to Therapeutic Targets. This figure illustrates the molecular pathways linking estrogen deficiency to bone loss and therapeutic intervention points. Arrows indicate the direction of signaling cascades and their downstream cellular effects. Color coding: Light blue represents the central initiating event (estrogen deficiency); Light pink/red indicates pro-resorptive pathways (RANKL/OPG axis and cytokine cascade) leading to increased osteoclast activity; Light green indicates the anti-anabolic sclerostin/Wnt pathway leading to decreased osteoblast function; Darker pink/salmon shows cellular consequences (osteoclast activation and T-cell activation); Darker green shows osteoblast suppression; Yellow represents net bone loss; Dark blue-gray indicates therapeutic targets with corresponding drug classes. Abbreviations: IL, interleukin; M-CSF, macrophage colony-stimulating factor; OPG, osteoprotegerin; PTH, parathyroid hormone; PTHrP, PTH-related protein; RANKL, receptor activator of nuclear factor-κB ligand; SOST, sclerostin gene; TNF, tumor necrosis factor.

Osteocytes are the primary source of sclerostin, a glycoprotein that inhibits the Wnt signaling pathway essential for osteoblast differentiation, proliferation, and function. Wnt signaling promotes bone formation by stimulating osteoblast activity and suppressing osteoblast apoptosis. Sclerostin binds to LRP5/6 co-receptors and blocks Wnt ligand binding, thereby suppressing bone formation. In estrogen-deficient states, altered sclerostin regulation contributes to impaired bone formation capacity, creating an imbalance where resorption exceeds the skeleton’s ability to replace lost bone. (Figure 2). This mechanism forms the basis for romosozumab, a sclerostin-inhibiting antibody that stimulates bone formation [13].

### 2.4. Temporal Phases of Postmenopausal Bone Loss

Bone loss following menopause occurs in two distinguishable phases. The early accelerated phase lasts 5–10 years and is driven primarily by estrogen deficiency. It affects trabecular bone preferentially due to its higher surface-to-volume ratio and more rapid remodeling. Annual losses of 2–3% at the lumbar spine and 1–2% at the hip characterize this phase. This trabecular predominance explains the earlier occurrence of vertebral fractures in postmenopausal osteoporosis [14].

The subsequent slow phase begins approximately 10 years after menopause and continues indefinitely. It reflects residual estrogen deficiency, age-related calcium/vitamin D changes, secondary hyperparathyroidism, declining physical activity, reduced osteoblast function, and increased marrow adiposity. This phase affects both trabecular and cortical compartments. Cortical thinning and porosity contribute substantially to hip fracture risk in the elderly, explaining the exponential increase in hip fracture incidence after age 70 [15].

### 2.5. Genetic and Epigenetic Factors

While estrogen deficiency represents the primary driver of postmenopausal bone loss, genetic factors account for 50–85% of the variance in peak bone mass and significantly influence fracture susceptibility. Genome-wide association studies have identified over 500 loci associated with BMD and fracture risk, including variants in genes encoding RANKL, OPG, sclerostin (SOST), LRP5, vitamin D receptor, collagen type 1, and estrogen receptors. Rare monogenic disorders such as osteogenesis imperfecta, juvenile Paget disease, and sclerosteosis have illuminated critical bone regulatory pathways—indeed, the therapeutic target for romosozumab was identified through study of sclerosteosis patients lacking functional sclerostin [16].

Epigenetic mechanisms—DNA methylation, histone modifications, and non-coding RNAs—modulate gene expression in response to environmental stimuli including hormonal changes, mechanical loading, and aging. MicroRNAs such as miR-21, miR-133a, and miR-148a regulate osteoblast and osteoclast differentiation and have emerged as potential biomarkers and therapeutic targets. Age-related changes in DNA methylation patterns at key bone-regulatory loci may contribute to the impaired osteoblast function characteristic of senile osteoporosis. The interplay between genetic predisposition and epigenetic modulation helps explain why individuals with similar estrogen exposure may exhibit markedly different rates of bone loss and fracture risk. Understanding these factors may eventually enable personalized risk stratification and targeted therapeutics [17].

## 3. Diagnostic Evaluation

### 3.1. Clinical Assessment and Risk Factor Identification

Comprehensive diagnosis of postmenopausal osteoporosis integrates clinical risk factor assessment, bone mineral density measurement, vertebral fracture identification, and exclusion of secondary causes. The clinical evaluation should systematically identify established risk factors that independently predict fracture risk beyond bone density measurements. Major risk factors recognized by FRAX and other risk assessment tools include advanced age, prior fragility fracture at any skeletal site (the strongest predictor of future fracture), parental history of hip fracture, current glucocorticoid use at doses equivalent to or exceeding 5 milligrams of prednisone daily for three or more months, rheumatoid arthritis and other inflammatory conditions, current tobacco smoking, and alcohol consumption exceeding three units daily [18].

Secondary causes of osteoporosis warrant systematic investigation, particularly in women presenting with unexpectedly low bone density for age, those with fractures occurring before sixty years of age, or those with Z-scores below −2.0. Important secondary causes include hyperthyroidism, primary hyperparathyroidism, celiac disease and other malabsorptive conditions, inflammatory bowel disease, chronic kidney disease with mineral and bone disorder, multiple myeloma and other hematological malignancies, systemic mastocytosis, and medication effects from aromatase inhibitors, proton pump inhibitors, anticonvulsants, and excess thyroid hormone replacement. Appropriate laboratory and clinical investigation should be guided by clinical suspicion and may reveal treatable underlying conditions [19].

Physical examination should assess height through serial measurements to detect occult vertebral fractures, with prospective height loss exceeding two centimeters or historical height loss exceeding four centimeters suggesting vertebral compression requiring imaging confirmation. Additional examination findings suggesting vertebral fractures include thoracic kyphosis, reduced rib-to-pelvis distance (less than two fingerbreadths), and increased occiput-to-wall distance during standing assessment against a wall. Low body weight (BMI below 20 kg/m^2^) and evidence of frailty or sarcopenia may indicate increased fracture risk [20].

### 3.2. Fracture Risk Assessment Tools

The Fracture Risk Assessment Tool (FRAX) represents the most widely validated and implemented algorithm for estimating ten-year probability of major osteoporotic fracture (hip, spine, proximal humerus, and distal forearm) and hip fracture specifically based on clinical risk factors with or without femoral neck bone mineral density. Country-specific FRAX models, available for over 70 countries, account for regional variations in fracture epidemiology and mortality. FRAX limitations include inability to capture dose–response relationships, exclusion of lumbar spine BMD, and potential underestimation of imminent fracture risk following recent fracture [21,22]. A persistent controversy surrounds T-score-based versus FRAX-based intervention thresholds, with major guidelines diverging significantly. The Endocrine Society recommends treatment at T-score ≤ −2.5 or FRAX thresholds of ≥3% for hip and ≥20% for major osteoporotic fracture, while the USPSTF focuses primarily on FRAX-based screening for women over 65 years. This discrepancy creates clinical uncertainty: a 70-year-old woman with T-score −2.3 and no other risk factors may qualify for treatment by some guidelines but not others. We contend that rigid adherence to either approach is suboptimal; instead, clinicians should integrate T-score, FRAX probability, and clinical judgment—particularly for patients near threshold values where factors not captured by FRAX (fall history, frailty, recent fracture timing) may tip the balance toward treatment. The arbitrary nature of thresholds must be acknowledged: fracture risk is continuous, and the difference between a 19% and 21% FRAX probability is clinically negligible.

Other fracture-risk assessment tools are available. The Garvan Bone Fracture Risk Calculator provides 5- and 10-year estimates of osteoporotic fracture risk and uniquely incorporates fall history into its algorithm. The American Bone Health Fracture Risk Calculator, developed by the Bone Health and Osteoporosis Foundation, includes a broader range of clinical risk factors than FRAX. QFracture, which does not require BMD measurements, can estimate fracture risk over any time interval between 1 and 10 years.

The Trabecular Bone Score (TBS), derived from texture analysis of lumbar spine DXA images, provides information regarding bone microarchitecture independent of bone mineral density and can be incorporated into FRAX calculations through published adjustment factors. TBS values correlate with trabecular number, separation, and connectivity. TBS below 1.23 indicates degraded microarchitecture, values above 1.31 indicate normal structure, and intermediate values suggest partial degradation. TBS is particularly valuable in conditions where BMD underestimates fragility including diabetes mellitus and glucocorticoid therapy [21,22]. However, the real-world utility of TBS warrants critical appraisal. TBS is not universally available, requires specific software licensing, and adds cost without strong evidence that TBS-adjusted treatment decisions improve fracture outcomes compared to BMD-based decisions alone. The FRAX adjustment for TBS is modest—typically shifting 10-year probability by 1–3 percentage points—and may not change management for patients clearly above or below intervention thresholds. We suggest TBS is most valuable in borderline cases where BMD-based FRAX falls near the treatment threshold, particularly in patients with diabetes or glucocorticoid exposure where BMD systematically underestimates risk. Routine TBS measurement for all patients cannot be justified by current evidence [23].

### 3.3. Laboratory Evaluation

Baseline laboratory assessment serves dual purposes: excluding secondary causes of bone loss and establishing parameters for subsequent treatment monitoring. Recommended initial investigations include complete blood count (to screen for hematological malignancy), serum calcium (elevated in hyperparathyroidism, malignancy), phosphate, alkaline phosphatase (elevated in osteomalacia, Paget disease), renal function assessment with eGFR, 25-hydroxyvitamin D (target above 50 nmol/L), and thyroid-stimulating hormone. Additional investigations based on clinical suspicion may include serum protein electrophoresis, 24 h urinary calcium excretion, intact parathyroid hormone, celiac serology (tissue transglutaminase antibodies), and assessment for hypercortisolism [24].

### 3.4. Systematic Clinical Evaluation: What to Ask and Assess

A systematic approach to evaluating patients with suspected osteoporosis ensures comprehensive assessment of fracture risk and identification of secondary causes. The following table summarizes the key elements of history, physical examination, and investigations that should be obtained in all patients presenting for osteoporosis evaluation (Table 1).

## 4. Bone Mineral Density Evaluation: When to Perform DXA

### 4.1. Indications for Initial DXA Screening

Dual-energy X-ray absorptiometry (DXA) screening enables identification of individuals with low bone mass before fractures occur, allowing implementation of preventive strategies. However, universal screening of all postmenopausal women is not recommended due to cost-effectiveness considerations. Instead, targeted screening based on age and risk factors optimizes resource utilization while identifying those most likely to benefit from intervention. Major professional organizations including the International Society for Clinical Densitometry, National Osteoporosis Foundation, Endocrine Society, and American Association of Clinical Endocrinologists have published screening recommendations that guide clinical practice [25,26] (Table 2).

Age-based screening recommendations center on the recognition that fracture risk increases exponentially with age and that the yield of screening improves substantially in older populations. DXA screening is recommended for all women aged 65 years and older regardless of clinical risk factors, reflecting the high prevalence of osteoporosis and elevated absolute fracture risk in this age group. For postmenopausal women younger than 65 years, DXA is recommended when clinical risk factors are present that increase fracture probability to a level comparable to that of a 65-year-old woman without additional risk factors. Risk assessment tools such as FRAX without BMD or the Osteoporosis Self-Assessment Tool can help identify younger postmenopausal women who warrant screening [27].

### 4.2. Frequency of Repeat DXA Testing

The optimal interval for repeat DXA testing depends on clinical context, baseline BMD, treatment status, and the precision of the measurement. For patients on osteoporosis treatment, repeat DXA is typically performed after one to two years to assess treatment response, with subsequent monitoring every two years if stable. The expected BMD change should exceed the least significant change (LSC) of the measurement facility to be considered clinically meaningful. For untreated patients with osteopenia, monitoring intervals can be extended based on baseline T-score: patients with T-scores between −1.5 and −2.0 may be reassessed every three to five years, while those with T-scores between −2.0 and −2.5 warrant more frequent monitoring every two to three years [24].

### 4.3. Limitations of DXA and Practical Adaptations

**Figure 3:**
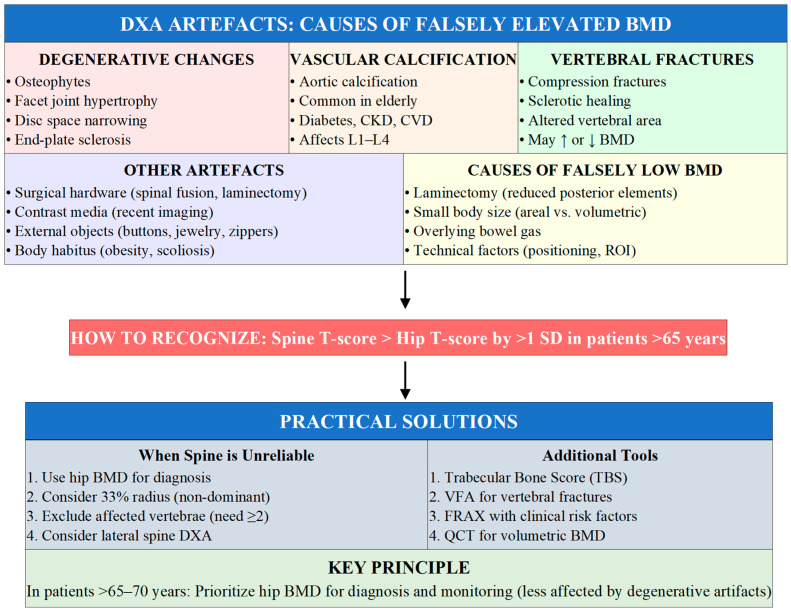
Common Artefacts and Interpretation Pitfalls in DXA Measurement. This figure illustrates the common sources of DXA measurement error and practical solutions for clinical interpretation. Arrows indicate the diagnostic workflow from artifact recognition to practical solutions and key principles. Color coding: Dark blue header indicates the main topic (causes of falsely elevated BMD); Light pink/rose boxes identify specific causes of falsely elevated BMD (degenerative changes, vascular calcification, vertebral fractures); Light blue boxes indicate other artefacts and causes of falsely low BMD; Red/coral box highlights the clinical recognition criterion (spine T-score > hip T-score by >1 SD); Yellow box presents practical solutions when spine measurements are unreliable; Green box lists additional diagnostic tools; Dark blue-gray box at bottom contains the key clinical principle for patients > 65–70 years. Abbreviations: BMD, bone mineral density; CKD, chronic kidney disease; CVD, cardiovascular disease; QCT, quantitative computed tomography; ROI, region of interest; SD, standard deviation; TBS, trabecular bone score; VFA, vertebral fracture assessment. DXA artifact recognition and practical adaptations from Blake & Fogelman [30] and Lewiecki et al. [31]. TBS utility from Silva et al. [22].

Despite its clinical utility, DXA has important inherent limitations that clinicians must recognize. DXA provides areal bone mineral density expressed as grams per square centimeter rather than true volumetric density, resulting in size-dependent measurements that underestimate BMD in smaller individuals (Figure 3). Degenerative spine disease, aortic calcification, and vertebral fractures can falsely elevate lumbar spine BMD, potentially masking true osteoporosis. These artifacts increase with age, making hip measurements more reliable for diagnosis in elderly patients. When spine and hip T-scores are discordant by more than one standard deviation with the spine appearing better preserved, degenerative artifact should be suspected [28,29].

Practical adaptations to address DXA limitations include rigorous quality assessment with exclusion of vertebrae affected by focal abnormalities, prioritizing hip measurements in patients over 65–70 years, utilizing the 33% radius when central sites are invalid, integrating Trabecular Bone Score for microarchitectural information, performing vertebral fracture assessment (VFA) to identify fractures that alter management, and incorporating clinical risk factors through FRAX rather than relying on BMD alone. When standard DXA cannot provide reliable assessment, quantitative CT may be considered for volumetric BMD measurement [30,31]. Quantitative computed tomography (QCT) provides true volumetric BMD (mg/cm^3^) and separates trabecular from cortical bone, overcoming several DXA limitations. QCT is particularly valuable when DXA is unreliable due to severe degenerative disease, vertebral fractures, or morbid obesity, and in patients with short stature where areal BMD systematically underestimates bone strength. However, QCT involves higher radiation exposure (50–300 μSv vs. 1–6 μSv for DXA), greater cost, and less standardized reference databases. FRAX does not incorporate QCT-derived BMD, limiting its integration with fracture probability algorithms. In clinical practice, QCT should be reserved for specific indications: (1) when DXA is technically inadequate at both spine and hip; (2) when discordance between clinical fractures and DXA T-scores suggests BMD underestimation; (3) for monitoring in patients with very high body mass index; and (4) when trabecular/cortical compartment information would alter management [32].

Cost-effectiveness and access to DXA varies substantially across healthcare settings, creating disparities in osteoporosis detection and treatment. In well-resourced health systems, DXA is widely available with costs of $100–250 per scan; in many low- and middle-income countries, DXA availability is limited to major urban centers, costs may be prohibitive without insurance coverage, and long waiting times delay diagnosis. This access inequality perpetuates the treatment gap: regions with lowest DXA availability often have highest fracture-related mortality. Alternative approaches—including FRAX without BMD, calcaneal quantitative ultrasound as a screening tool, and opportunistic CT-based BMD assessment from abdominal imaging—may expand screening capacity in resource-limited settings. Healthcare systems should consider targeted DXA allocation to highest-risk individuals identified by clinical risk factors when universal access is not feasible [33].

## 5. Treatment Strategies

### 5.1. Treatment Indications and Risk Stratification

Treatment decisions should integrate BMD measurements, fracture history, and estimated fracture probability. Indications for pharmacotherapy include: T-score ≤ −2.5 at spine, femoral neck, or total hip; T-score −1.0 to −2.5 with hip or spine fragility fracture; T-score −1.0 to −2.5 with FRAX 10-year hip fracture probability ≥ 3% or major osteoporotic fracture ≥ 20% (thresholds vary by country); and low bone mass with high-risk factors such as glucocorticoid therapy [27]. Before detailing pharmacological options, it is essential to emphasize that comprehensive fracture prevention requires addressing both bone fragility and fall risk from the outset of treatment planning. Over 90% of hip fractures result from falls; therefore, fall risk assessment should be performed alongside fracture risk evaluation in all patients. Patients identified as high fall risk (history of ≥2 falls in past year, gait/balance impairment, Timed Up and Go > 12 s) should receive concurrent fall prevention interventions—exercise programs, medication review, home safety assessment—as detailed in Section 5.4. The most effective osteoporosis pharmacotherapy cannot prevent a fracture from a fall that would break even normally dense bone. Fall prevention and pharmacotherapy are complementary, not alternative, strategies.

**Figure 4:**
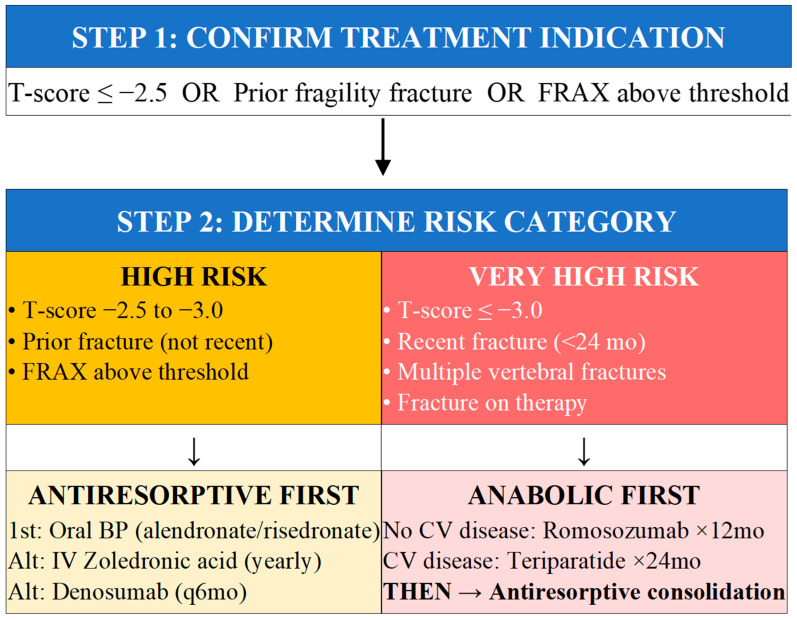
Therapeutic Decision Algorithm for Postmenopausal Osteoporosis: Treatment Selection Based on Fracture Risk Stratification. This figure presents a stepwise algorithm for selecting osteoporosis treatment based on fracture risk stratification. Arrows indicate the sequential decision-making process from treatment indication confirmation through risk categorization to specific treatment recommendations. Color coding: Blue boxes indicate step headers and key decision points (Step 1: treatment indication; Step 2: risk category determination); Yellow box identifies high-risk patients and corresponding antiresorptive-first treatment approach; Red/coral box identifies very high-risk patients requiring anabolic-first therapy; Light yellow box shows antiresorptive treatment options for high-risk patients; Light pink box shows anabolic treatment options for very high-risk patients with subsequent antiresorptive consolidation. The algorithm emphasizes that very high-risk patients benefit from initial anabolic therapy followed by antiresorptive consolidation, while high-risk patients may begin with antiresorptive agents. Abbreviations: BP, bisphosphonate; CV, cardiovascular; IV, intravenous; mo, months. Risk stratification criteria from Kanis et al. [34] and Johansson et al. [35]. Treatment sequencing recommendations from Shoback et al. [36] and Cosman et al. [37].

Risk stratification increasingly guides not only the decision to treat but also the selection of initial therapy. The concept of very high or imminent fracture risk identifies patients who may benefit from more potent initial therapy with osteoanabolic agents, including those with recent fracture within the preceding 24 months (particularly hip or vertebral), very low bone density with T-score at or below negative 3.0, multiple prevalent vertebral fractures, fractures occurring despite antiresorptive therapy, very high FRAX probability (major osteoporotic fracture above 30% or hip fracture above 4.5%), and high-dose glucocorticoid therapy [34,35]. As summarized in Table 3, the fracture reduction profiles differ substantially between drug classes, which directly informs the risk-adapted algorithm presented in Figure 4. Antiresorptives (bisphosphonates, denosumab) demonstrate consistent vertebral fracture reduction of 40–70% but variable non-vertebral and hip fracture efficacy. Anabolics (teriparatide, romosozumab) achieve superior vertebral protection and, for romosozumab, significant hip fracture reduction—justifying their prioritization in very high-risk patients where rapid BMD accrual and fracture prevention are paramount. The mechanism column in Table 3 should be read in conjunction with Figure 2 (molecular pathways): each drug class targets a specific node in the estrogen deficiency cascade, making drug selection a logical extension of understanding pathophysiology rather than an arbitrary guideline recommendation.

### 5.2. Algorithm for Initial Treatment Selection

Selection of initial therapy should follow a systematic algorithm matching treatment intensity to fracture risk severity. For high-risk patients without very high-risk features, oral bisphosphonates represent appropriate first-line therapy given extensive efficacy data, favorable safety profile, and low cost. Intravenous zoledronic acid or denosumab are alternatives when oral therapy is contraindicated or adherence is concerning. For very high-risk patients, osteoanabolic-first strategies produce greater BMD gains and potentially superior fracture reduction: romosozumab for 12 months (if no cardiovascular contraindication) or teriparatide/abaloparatide for up to 24 months, followed by antiresorptive consolidation [36,37]. Critical appraisal of the ‘very high risk’ category reveals important nuances that guidelines often gloss over. The criteria—T-score ≤ −3.0, recent fracture within 24 months, multiple vertebral fractures—are not equivalent in their implications. A patient with T-score −3.2 but no prior fracture has fundamentally different biology than a patient with T-score −2.6 and two recent vertebral fractures; the latter has demonstrated skeletal fragility beyond what BMD predicts. We argue that recent fracture should carry greater weight in treatment intensification decisions than T-score threshold alone. Furthermore, ‘fracture on therapy’ conflates bisphosphonate failure (suggesting need for alternative mechanism) with suboptimal adherence (suggesting need for adherence intervention rather than drug escalation).

The cost-effectiveness of anabolic-first strategies represents a significant barrier to implementation that deserves frank discussion. Romosozumab costs approximately $22,000–25,000 annually in the United States; teriparatide similarly ranges $20,000–30,000 per year. By contrast, generic alendronate costs under $100 annually. While modeling studies suggest anabolic-first approaches may be cost-effective in very high-risk patients when subsequent fractures and their costs are considered, real-world access remains problematic. Many healthcare systems restrict anabolic agents to patients who have failed bisphosphonates—a sequencing that contradicts the evidence supporting anabolic-first strategies. Clinicians must navigate these access barriers while advocating for evidence-based sequencing. For patients unable to access anabolic therapy, denosumab or zoledronic acid represent reasonable alternatives that provide rapid, potent antiresorptive effect [38].

### 5.3. Non-Pharmacological Interventions

Adequate calcium (1000–1200 mg daily, preferably dietary) and vitamin D (800–2000 IU daily, targeting 25-hydroxyvitamin D ≥ 50 nmol/L) form the foundation of osteoporosis management [39]. Exercise should emphasize weight-bearing and resistance activities for bone and muscle strength, plus balance training for fall prevention. Smoking cessation and alcohol moderation support skeletal health. However, given that over 90% of hip fractures result from falls, comprehensive fall prevention represents a critical and often underemphasized component of fracture risk reduction [40,41,42,43,44].

### 5.4. Fall Prevention: A Critical Component of Fracture Risk Reduction

Falls represent the proximate cause of the vast majority of osteoporotic fractures, particularly hip fractures. Approximately one-third of community-dwelling adults aged 65 and older fall each year, with the rate increasing to 50% among those over 80. The combination of reduced bone strength from osteoporosis and increased fall propensity from aging creates a multiplicative increase in fracture risk. While pharmacological therapy addresses bone fragility, fall prevention strategies address the mechanical event that precipitates fracture. Comprehensive fracture prevention therefore requires attention to both components [40,41,42,43,44,45,46,47,48] (Table 4).

#### 5.4.1. Fall Risk Assessment

All patients with osteoporosis should undergo systematic fall risk assessment. A history of falls in the preceding 12 months is the strongest predictor of future falls; patients reporting two or more falls, or one fall with injury, warrant comprehensive evaluation. The Timed Up and Go test provides a simple office-based assessment: the patient rises from a standard chair, walks 3 m, turns, walks back, and sits down. Times exceeding 12 s indicate increased fall risk and impaired mobility. Additional assessment should evaluate gait pattern (shuffling, asymmetry, wide-based), balance (tandem stance, single leg stance), lower extremity strength (chair rise without arms), and cognitive function (as cognitive impairment increases fall risk substantially) [40,41].

#### 5.4.2. Medication Review

Polypharmacy and specific medication classes substantially increase fall risk. Medications warranting particular scrutiny include: sedatives and hypnotics (benzodiazepines, Z-drugs), which impair balance and reaction time; antihypertensives, particularly those causing orthostatic hypotension (alpha-blockers, diuretics, vasodilators); psychotropic medications (antidepressants, antipsychotics, anxiolytics); opioid analgesics; anticonvulsants; and anticholinergic drugs. The use of four or more medications from any class (polypharmacy) independently increases fall risk. Medication review should aim to discontinue unnecessary drugs, reduce doses where possible, substitute safer alternatives, and time administration to minimize impact (e.g., taking diuretics in the morning rather than evening) [42].

#### 5.4.3. Vision Assessment and Correction

Visual impairment significantly increases fall risk through multiple mechanisms: reduced detection of environmental hazards, impaired depth perception, and compromised balance (vision contributes to postural stability). All patients should have regular ophthalmological evaluation with correction of refractive errors. Cataracts should be treated surgically when vision is significantly impaired. Importantly, multifocal lenses (bifocals, progressive lenses), while convenient, impair depth perception and edge-contrast sensitivity when walking; patients at high fall risk may benefit from single-vision distance glasses for outdoor mobility, reserving multifocals for reading and desk work. Home lighting should be optimized, with night lights in hallways and bathrooms [43].

#### 5.4.4. Exercise and Physical Therapy

Exercise represents one of the most effective fall prevention interventions, with meta-analyses demonstrating 20–40% reduction in fall rates with appropriate programs. The most effective programs incorporate multiple components: balance training (tai chi, standing on one leg, tandem walking), strength training (particularly lower extremity), and gait training. Tai chi has particularly strong evidence, with studies showing 40–50% reduction in falls among older adults. Exercise programs should be individualized based on baseline function; for frail individuals, physical therapy referral for supervised training is appropriate. Programs should be sustained, as benefits diminish when exercise stops. Walking alone, while beneficial for general health, does not reduce falls and may increase fall exposure without improving balance [44,45].

#### 5.4.5. Home Safety Modifications

Environmental hazards contribute to approximately 30–50% of falls in older adults. Home safety assessment, ideally performed by an occupational therapist, identifies modifiable risks. Key interventions include: removing loose rugs and floor clutter; ensuring adequate lighting throughout the home, especially in stairways, hallways, and bathrooms; installing grab bars near toilets and in bathtubs/showers; using non-slip mats in bathrooms; securing handrails on all stairs; arranging furniture to create clear pathways; keeping frequently used items within easy reach to avoid climbing; and addressing uneven surfaces or thresholds. For patients with significant mobility impairment, more extensive modifications such as stairlifts, walk-in showers, or single-floor living arrangements may be warranted [45].

#### 5.4.6. Footwear

Inappropriate footwear contributes to falls, yet is often overlooked in clinical assessments. High-risk footwear includes high heels (>2.5 cm), shoes with slippery soles, loose-fitting slippers, and walking barefoot or in stockinged feet. Optimal footwear for fall prevention features: low heels (<2.5 cm), firm heel counters (supporting the heel), thin, firm, slip-resistant soles that allow proprioceptive feedback, and secure fastening (laces, velcro). Patients should be counseled against wearing slippers without backs or walking without shoes, particularly on hard or slippery surfaces. For patients with foot deformities or neuropathy, referral to podiatry for custom orthotic assessment may be beneficial [46].

#### 5.4.7. Management of Orthostatic Hypotension

Orthostatic hypotension, defined as a drop in systolic blood pressure ≥ 20 mmHg or diastolic ≥ 10 mmHg within 3 min of standing, affects 20–30% of older adults and significantly increases fall risk. Assessment should include standing blood pressure measurement in all patients with falls or dizziness. Management strategies include: reviewing and reducing offending medications; advising patients to rise slowly from lying or sitting (pause at bed edge before standing); encouraging adequate hydration (1.5–2 L daily unless contraindicated); considering compression stockings (thigh-high provide better effect than knee-high); elevating the head of the bed 10–15 degrees to reduce nocturnal natriuresis; and avoiding large meals and alcohol, which can exacerbate postprandial and generalized hypotension. For refractory cases, pharmacological treatment with fludrocortisone or midodrine may be considered [47].

#### 5.4.8. Vitamin D for Fall Prevention

Beyond its role in bone health, vitamin D has direct effects on muscle function, and deficiency is associated with increased fall risk. Vitamin D receptors are present in skeletal muscle, and vitamin D status correlates with muscle strength and function, particularly in the lower extremities. Meta-analyses suggest that vitamin D supplementation reduces fall risk by approximately 14–20%, with greater benefits in those who are deficient at baseline. The effect appears most consistent with doses of 700–1000 IU daily achieving serum 25-hydroxyvitamin D levels above 60 nmol/L (24 ng/mL). Very high intermittent doses (annual bolus injections) may paradoxically increase fall risk and should be avoided. For osteoporosis patients, ensuring vitamin D sufficiency serves the dual purpose of optimizing bone health and reducing fall propensity [48].

## 6. Medication-Related Osteonecrosis of the Jaw (MRONJ)

### 6.1. Definition and Clinical Presentation

Medication-related osteonecrosis of the jaw (MRONJ) is defined as exposed bone or bone that can be probed through an intraoral or extraoral fistula in the maxillofacial region, persisting for more than eight weeks, in patients currently or previously treated with antiresorptive or antiangiogenic agents, and with no history of radiation therapy to the jaws or obvious metastatic disease to the jaws. Clinical presentation varies widely, ranging from asymptomatic exposed bone discovered incidentally to severe pain, soft tissue swelling, purulent discharge, pathological fracture, and orocutaneous fistula formation requiring extensive surgical intervention [49].

MRONJ staging guides both prognosis and management. Stage 0 involves no clinical evidence of necrotic bone but presence of non-specific symptoms such as jaw pain or odontalgia not explained by dental pathology, or radiographic findings such as alveolar bone loss or sclerosis. Stage 1 presents with exposed necrotic bone or fistula probing to bone in asymptomatic patients without evidence of infection. Stage 2 demonstrates exposed necrotic bone with evidence of infection including pain, erythema, with or without purulent drainage. Stage 3 includes exposed necrotic bone with pain, infection, and one or more of: pathological fracture, extraoral fistula, oral-antral or oral-nasal communication, or osteolysis extending to the inferior mandibular border or sinus floor [50].

### 6.2. Pathophysiology

The pathophysiology of MRONJ is multifactorial and incompletely understood, involving the unique characteristics of jaw bone physiology combined with antiresorptive drug effects and local precipitating factors. Several mechanisms have been proposed to explain why the jaw is preferentially affected while other skeletal sites are spared [51].

Suppressed bone remodeling represents the primary mechanism. Bisphosphonates and denosumab potently inhibit osteoclast-mediated bone resorption, which is essential for normal bone remodeling and repair of accumulated microdamage. The jaw bones, particularly the alveolar processes, have the highest remodeling rates in the skeleton due to constant mechanical stress from mastication and the presence of teeth. This high turnover results in greater drug accumulation in jaw bone for bisphosphonates and greater sensitivity to remodeling suppression. When remodeling is profoundly suppressed, the jaw loses its capacity to repair microdamage from normal function and to heal following dental procedures [52].

Impaired angiogenesis contributes to MRONJ pathogenesis. Bisphosphonates, particularly nitrogen-containing compounds, have antiangiogenic properties that impair blood vessel formation and endothelial cell function. The jaw has a relatively tenuous blood supply compared to long bones, making it more vulnerable to ischemic injury. Reduced vascularity compromises healing capacity following trauma or dental procedures. Infection with biofilm formation perpetuates the process, with Actinomyces species particularly prominent in MRONJ lesions. Once established, biofilm infection creates a self-sustaining cycle of inflammation and bone destruction [53].

### 6.3. Incidence and Risk Factors

The incidence of MRONJ varies dramatically based on clinical setting, drug type, dose, and duration. In osteoporosis patients receiving oral bisphosphonates, the estimated incidence is approximately 1 per 10,000 to 1 per 100,000 patient-years—similar to the background incidence of osteonecrosis of the jaw in the general population. For patients receiving intravenous zoledronic acid for osteoporosis (5 mg annually), incidence remains low at approximately 1 per 10,000 to 1 per 50,000 patient-years. Denosumab 60 mg every 6 months for osteoporosis carries similar risk to bisphosphonates [54].

In contrast, cancer patients receiving high-dose antiresorptive therapy face substantially higher risk. Monthly IV zoledronic acid (4 mg) for bone metastases carries MRONJ incidence of 1–2%, while denosumab 120 mg monthly may reach 1–5%. This 100-fold difference between osteoporosis and oncology dosing underscores the importance of drug exposure. Major risk factors include: drug-related (high dose, long duration); dental (tooth extraction precipitates 50–60% of cases, periodontal disease, implants); systemic (glucocorticoids, chemotherapy, diabetes); and anatomical (mandible affected twice as often as maxilla) [55].

### 6.4. Prevention Strategies

Prevention of MRONJ relies on risk assessment, dental optimization before initiating therapy, maintenance of oral health during treatment, and careful management of dental procedures. Before initiating antiresorptive therapy, particularly for cancer indications, patients should undergo comprehensive dental evaluation to identify teeth with poor prognosis requiring extraction, active periodontal disease, dental abscesses, and ill-fitting dentures. All necessary invasive dental procedures should be completed and healing allowed (minimum 2–3 weeks, ideally 4–6 weeks) before starting therapy [56,57,58,59].

For osteoporosis patients beginning treatment, formal pre-treatment dental clearance is not mandatory given the low absolute risk, but patients should be counseled to maintain regular dental care and inform their dentist of their medications. Ongoing oral hygiene maintenance is essential for all patients on antiresorptive therapy, including regular dental visits every 6–12 months, daily brushing and flossing, prompt treatment of caries to avoid extraction, and proper denture fit. Patients should be educated about MRONJ symptoms and instructed to report jaw pain, swelling, exposed bone, or non-healing extraction sites promptly [57,58].

When invasive dental procedures become necessary during therapy, careful planning minimizes risk. For osteoporosis patients on standard-dose therapy, routine procedures including extractions can generally proceed without drug modification. The concept of drug holidays before dental procedures remains controversial for bisphosphonates given their prolonged skeletal retention. When extractions are performed, techniques to minimize MRONJ risk include atraumatic extraction, alveoloplasty to eliminate sharp bone edges, primary wound closure when possible, perioperative antibiotics, chlorhexidine rinses, and close follow-up. Critically, the benefit–risk ratio strongly favors osteoporosis treatment: MRONJ risk is approximately 1 in 10,000–100,000 while hip fracture risk without treatment approaches 1 in 6 for women [57,58].

### 6.5. Addressing Dental Anxiety in Patients and Healthcare Providers

A significant barrier to both osteoporosis treatment adherence and appropriate dental care is the disproportionate anxiety surrounding MRONJ among patients and dental practitioners. This anxiety often leads to two problematic scenarios: patients refusing or discontinuing effective osteoporosis treatment due to fear of jaw complications, and dentists refusing to perform necessary dental procedures in patients on antiresorptive therapy. Both situations ultimately harm patients—the former by leaving osteoporosis untreated with its attendant fracture risk, the latter by allowing dental disease to progress to the point where more invasive intervention becomes necessary, paradoxically increasing MRONJ risk [57,58,59,60].

#### 6.5.1. Contextualizing the Risk: Putting MRONJ in Perspective

For osteoporosis patients on standard-dose antiresorptive therapy, the absolute risk of MRONJ is exceedingly low and must be communicated in context. The incidence of approximately 1 per 10,000 to 1 per 100,000 patient-years is comparable to or lower than many accepted medical risks: the risk of serious gastrointestinal bleeding from low-dose aspirin (1 per 1000 per year), the risk of venous thromboembolism from oral contraceptives (1 per 1000 per year), or the background incidence of osteonecrosis of the jaw in the general population not taking any bone medications. In practical terms, a patient would need to be treated for 10,000 to 100,000 years to expect one case of MRONJ. Even with tooth extraction—the highest-risk dental procedure—the absolute risk increases only modestly, remaining well below 1% in osteoporosis patients [59].

Conversely, the consequences of untreated osteoporosis are substantial and well-documented. A 50-year-old woman has a lifetime hip fracture risk of approximately 17% (1 in 6), and hip fractures carry 20–25% one-year mortality. Vertebral fractures cause chronic pain, height loss, kyphotic deformity, and predict future fractures. The number needed to treat to prevent one fracture with bisphosphonates is approximately 15–20 over 3–5 years for high-risk patients. Thus, refusing to treat osteoporosis due to MRONJ fear exposes patients to a far greater and more certain harm than the remote possibility of jaw complications [59,60,61].

#### 6.5.2. Guidance for Dental Practitioners

Dental practitioners should understand that refusing to treat osteoporosis patients on antiresorptive therapy is not supported by evidence and may cause harm by allowing dental pathology to worsen. Professional dental organizations including the American Dental Association have issued guidance confirming that routine dental care, including extractions, can proceed in osteoporosis patients taking standard-dose bisphosphonates or denosumab. The key distinction that must be made is between osteoporosis dosing (oral bisphosphonates, zoledronic acid 5 mg annually, denosumab 60 mg every 6 months) and oncology dosing (monthly IV bisphosphonates, denosumab 120 mg monthly)—the latter carries substantially higher MRONJ risk and warrants more cautious approach [58].

For osteoporosis patients requiring dental extractions, the following approach is recommended. Drug holidays before dental procedures are generally NOT recommended for bisphosphonates, as these drugs persist in bone for years and brief interruption does not meaningfully reduce bone drug levels. For denosumab, some experts suggest timing procedures early in the dosing interval (1–2 months after injection) when bone turnover begins recovering, though evidence is limited. Surgical technique should emphasize atraumatic extraction, smoothing of sharp bony edges, primary closure when feasible, and perioperative antibiotics (amoxicillin or clindamycin). Chlorhexidine rinses before and after the procedure reduce bacterial load. Close follow-up at 1–2 weeks to assess healing is prudent. With these precautions, the vast majority of extractions heal uneventfully [62,63].

#### 6.5.3. Reassuring Patients: Key Messages

When counseling patients about MRONJ risk, clinicians should emphasize several key points. First, the risk is very low—comparable to many everyday risks that we accept without concern. Second, the risk applies primarily to cancer patients receiving much higher doses; osteoporosis doses are far safer. Third, good oral hygiene and regular dental care actually reduce MRONJ risk by preventing the dental infections and extractions that precipitate most cases. Fourth, if MRONJ does occur in osteoporosis patients, it is typically mild (Stage 1 or 2) and manageable with conservative measures; severe cases requiring surgery are rare. Fifth, the alternative—not treating osteoporosis—carries far greater risk of fractures, disability, and death. Patients should be encouraged to maintain excellent oral hygiene, see their dentist regularly, and inform dental providers of their medications, but should not avoid necessary dental care or refuse beneficial osteoporosis treatment [62,63,64].

#### 6.5.4. Communication Between Physicians and Dentists

Effective communication between prescribing physicians and dental providers is essential for optimal patient care. Physicians should provide patients with written documentation of their bone medication, dose, duration, and indication (osteoporosis versus cancer) to share with dental providers. When dentists have questions about proceeding with treatment, direct physician-to-dentist communication can clarify the low-risk nature of osteoporosis dosing and provide reassurance. Both physicians and dentists should avoid using language that amplifies patient anxiety, such as referring to MRONJ as ‘bone death’ or suggesting that dental work is ‘dangerous.’ Instead, emphasis should be placed on the importance of maintaining dental health, which actually reduces MRONJ risk by preventing the conditions that precipitate it [59,60,61,62,63].

## 7. When and How to Stop Antiresorptive Therapy

### 7.1. Rationale for Considering Discontinuation

Osteoporosis is a chronic disease requiring long-term treatment, yet the decision regarding treatment discontinuation requires careful individualization based on residual fracture risk, treatment type, duration, and patient preferences. The most concerning adverse events associated with both bisphosphonates and denosumab are atypical femoral fractures (AFFs) and osteonecrosis of the jaw (ONJ), both believed to stem from prolonged suppression of bone remodeling. Both events have an estimated overall incidence of 1 in 1000 to 1 in 10,000 in patients treated for osteoporosis, though incidence increases with treatment duration [64].

The risk-benefit balance of bisphosphonates can be evaluated by number needed to treat (NNT) and number needed to harm (NNH). In White women, bisphosphonate use corresponds to an NNT for preventing hip fracture of approximately 350 and 170 at 5 and 10 years, respectively. After five years of treatment, the NNH to cause an AFF was 12,500 for all women; however, this decreased to 2630 after 10 years of continuous treatment. Notably, the NNH at 10 years was disproportionately lower among Asian women at only 424, indicating higher AFF rates in this population. Additional risk factors for AFF include reduced height and weight and glucocorticoid use. Importantly, AFF risk decreases rapidly after bisphosphonate discontinuation, highlighting the importance of drug holidays to mitigate long-term risks while maintaining fracture prevention benefits [64,65].

### 7.2. Atypical Femoral Fractures: Recognition and Management

Atypical femoral fractures (AFFs) are stress fractures occurring in the subtrochanteric or diaphyseal femur with characteristic radiographic features distinguishing them from typical osteoporotic hip fractures. The ASBMR Task Force criteria require location along the femoral diaphysis from just distal to the lesser trochanter to just proximal to the supracondylar flare, plus at least 4 of 5 minor features: transverse or short oblique orientation, non-comminuted or minimally comminuted, localized periosteal or endosteal thickening at the fracture site (beaking), complete fractures extend through both cortices with a medial spike, and incomplete fractures involve only the lateral cortex. Bilateral involvement occurs in up to 28% of cases [66,67,68,69,70,71,72,73].

The pathophysiology involves prolonged suppression of bone remodeling, which impairs the skeleton’s ability to repair accumulated microdamage. This leads to stress fracture initiation, typically at the lateral cortex where tensile forces concentrate. The lateral cortical thickening (beaking) represents a failed healing response. Risk increases with treatment duration: incidence rises from ~2 per 100,000/year with <2 years of bisphosphonate use to ~78 per 100,000/year with >8 years of use. Asian ethnicity confers 2–3 fold higher risk. Other risk factors include femoral bowing, shorter stature, glucocorticoid use, and proton pump inhibitors [73].

Clinical presentation typically involves prodromal thigh or groin pain weeks to months before complete fracture—a critical window for intervention. Any patient on long-term antiresorptive therapy presenting with thigh pain should undergo radiographic evaluation; if plain films are negative but suspicion remains, MRI or bone scan can detect incomplete fractures. Management of incomplete AFFs includes immediate bisphosphonate discontinuation, protected weight-bearing, calcium/vitamin D optimization, and consideration of prophylactic intramedullary nailing for fractures involving >50% cortical width or showing progression. Teriparatide may accelerate healing in incomplete fractures, though evidence is limited. For complete AFFs, intramedullary nailing is preferred over plate fixation due to better outcomes. The contralateral femur should be imaged, as bilateral involvement is common [72,73].

### 7.3. Bisphosphonate Drug Holidays

Bisphosphonates can be classified by their relative osteoclast inhibition potency, determined primarily by bone affinity. Among commonly used agents, zoledronic acid has the highest affinity to bone, while ibandronate and risedronate have the lowest. This differential bone affinity translates into differing ‘tail effects’ after discontinuation. Long-term extension studies of alendronate demonstrated that after approximately five years of therapy, lumbar spine BMD essentially plateaued and persisted even after the drug was stopped for five years. In contrast, hip BMD and bone turnover returned toward baseline over approximately five years. The impact on fracture risk is nuanced: overall, there was no significant difference in morphometric vertebral, nonvertebral, and hip fractures between those continuing treatment for 10 years versus those discontinuing after 5 years; however, there was a 55% reduction in clinical vertebral fractures with continued therapy, and patients at highest fracture risk saw preferential benefit from continuing treatment [68,69].

Risedronate exhibits a comparatively much shorter tail effect than alendronate. While bone turnover and BMD in alendronate-treated patients return to baseline over approximately five years, risedronate-treated patients revert to baseline within approximately one year after stopping, regardless of treatment duration. Zoledronic acid has an even longer tail effect; a single infusion can sustain BMD gains over baseline for up to 10 years and reduce clinical fracture risk. This difference has clinical implications: US Medicare data examining fracture risk during drug holidays in over 81,000 women showed that discontinuing alendronate for more than two years was associated with a 30% increased fracture risk compared to continued therapy. Hip fracture rates were approximately 18% higher in patients who discontinued risedronate compared to alendronate during extended drug holidays [70,71].

The 2016 ASBMR guidance on drug holidays recommends that high-risk patients (age > 70 years, hip T-score < −2.5, prior major osteoporotic fracture, or fracture while on therapy) may benefit from extended treatment for up to 10 years with oral bisphosphonates or 6 years with intravenous zoledronic acid. For patients at low or moderate fracture risk after initial treatment, a drug holiday of two to three years may be considered. Many experts recommend slightly longer drug holiday duration for zoledronic acid (18–24 months) compared to oral bisphosphonates (no more than 12–24 months for alendronate, and perhaps less for risedronate). During drug holidays, patients should undergo periodic reassessment including BMD monitoring; a decrease in BMD > 3% is considered significant. Bone turnover markers such as C-terminal telopeptide (CTX) can guide decisions about treatment reinitiation: a return to pretreatment baseline levels or an increase > 30% from end-of-treatment levels suggests therapy should be restarted [71].

### 7.4. Denosumab Discontinuation: The Rebound Phenomenon

Denosumab exhibits distinct effects on bone that create unique challenges for discontinuation. Unlike bisphosphonates, denosumab effects are rapidly reversible. On discontinuation, bone turnover increases markedly, often surpassing pretreatment levels. At 6 months after the last injection, CTX rises rapidly, frequently exceeding baseline values. BMD typically returns to pretreatment levels within 1–2 years, sometimes dropping below baseline, especially at the hip. The rebound likely results from hyperactivation of dormant preosteoclasts that accumulate during treatment. Additional mechanisms include depletion of OPG-producing cells, leaving RANKL unopposed, and a mechanostatic reset where accumulated bone mass exceeds mechanical demands [74,75,76,77].

Most concerning is the substantially increased risk of multiple vertebral fractures following denosumab discontinuation. The incidence of multiple vertebral fractures was as high as 10–15% within the first year after stopping denosumab, even in patients subsequently treated with oral bisphosphonates. Risk factors for vertebral fractures after discontinuation include history of prevalent vertebral fractures, longer duration of denosumab use (>3 years), greater gain in BMD during treatment, greater loss of hip BMD after stopping, and chronic kidney disease. The rapid and profound rebound in bone turnover leads to such excessive and uncoordinated remodeling that it can result in perforation of trabeculae, not just thinning, which may explain why multiple vertebral fractures can occur within months of the last dose even in patients who had achieved substantial BMD gains [77,78,79,80,81,82,83,84,85,86,87,88,89].

### 7.5. Exit Strategies After Denosumab

The European Calcified Tissue Society (ECTS) strongly recommends initiating alternative antiresorptive treatment when the next denosumab injection is due. The preferred approach is administering zoledronic acid 6 months after the final denosumab dose, when denosumab’s effects begin to wane. Timing is crucial: administration too late (>9 months) means peak rebound may have occurred; too early means less exposed bone surface for bisphosphonate binding. With prolonged denosumab use, rebound is more pronounced, and a single bisphosphonate dose may be insufficient [80]. A critical evidence gap must be acknowledged: the current recommendations for post-denosumab transition are largely extrapolated from observational data and expert consensus rather than randomized controlled trials specifically designed to evaluate fracture outcomes with different exit strategies. The ECTS guidelines are based on surrogate markers (CTX suppression, BMD maintenance) rather than demonstrated fracture reduction. No randomized trial has proven that zoledronic acid at 6 months prevents vertebral fractures better than alternative timing or agents. The optimal number of zoledronic acid doses, the role of oral bisphosphonates as alternatives, and the duration of post-transition monitoring remain empirically guided rather than evidence-proven. This uncertainty creates a clinical dilemma: clinicians must act decisively (given catastrophic rebound risk) while acknowledging the evidence base is weaker than for initial treatment selection. We advocate for shared decision-making that transparently communicates this uncertainty [82,84].

Monitoring bone turnover markers after denosumab discontinuation helps tailor subsequent therapy. For patients showing significant increases in markers, rapid BMD loss, or not responding to a single dose of zoledronic acid, additional doses may be required. If BTMs remain elevated at 6 months, clinicians could consider adding alendronate for 6 months before administering a second dose of zoledronic acid 12 months after denosumab discontinuation. An alternative approach involves gradual dose reduction (to 30 mg then 15 mg) which preliminary data suggest could prevent significant bone loss; a combined approach with reduced denosumab dose paralleled by zoledronic acid administration may eventually prove effective. For patients responding well to denosumab with improving BMD and no fractures, continuation remains a reasonable strategy as there is no compelling evidence that denosumab becomes less effective or more dangerous with extended use (tested through 10 years). For patients who fracture despite long-term denosumab, the best strategy may be adding an osteoanabolic medication rather than discontinuing [76].

Switching from denosumab to a PTH analog is not recommended, as the rebound effect could be amplified by stimulation of bone remodeling. Although switching from denosumab to romosozumab has been shown to partially attenuate rebound, vertebral fractures have been reported after this sequential treatment. When denosumab duration is limited, switching to romosozumab can increase lumbar spine BMD greater than continuing denosumab alone, though CTX rebound becomes evident within three months of the transition [86]. The key principle is that denosumab discontinuation should never occur without a planned exit strategy, and patients initiating denosumab should be counseled about this long-term commitment [81,83,84,85,86,87,88,89,90,91]. Several recently completed and ongoing trials have provided critical new evidence on treatment sequencing and discontinuation strategies through 202 [82,83,84,85,86,87,88,89,90,91]. The Denosumab Sequential Therapy (DST) randomized trial (Lee et al., JAMA Network Open 2024) demonstrated that a single zoledronic acid infusion at 6 months after denosumab discontinuation did not fully prevent lumbar spine BMD loss in patients treated with denosumab for ≥2 years, though femoral neck and total hip BMD were preserved—highlighting that current exit strategies may be insufficient for long-term denosumab users [87]. A 2024 Italian study (Grassi et al., JCEM 2024) evaluated repeated versus single zoledronic acid administration following ECTS guidelines, finding that patients with CTX ≥ 280 ng/L at 6 months benefited from a second zoledronic acid dose, though BMD loss still occurred in some patients [88]. The FRAME extension post hoc analysis (Cosman et al., JBMR 2024) confirmed that romosozumab followed by denosumab produces significantly greater BMD gains and higher probability of achieving T-score above −2.5 compared to denosumab alone—supporting anabolic-first sequencing in very high-risk patients [89]. Emerging data on overlapping romosozumab-denosumab strategies (Kumar et al., Osteoporos Int 2024) suggest that initiating romosozumab 3 months after last denosumab dose with early denosumab recommencement may maximize BMD gains while mitigating rebound, though larger controlled trials are needed [90]. Clinicians should incorporate these 2024 findings into treatment planning while awaiting longer-term fracture endpoint data. Several ongoing observational and prospective studies are evaluating sequential strategies after denosumab or anabolic-first approaches, although randomized trials powered for fracture outcomes remain lacking.

## 8. Conclusions

This review advances a simple but consequential thesis: the next breakthrough in postmenopausal osteoporosis will not come from discovering new drugs, but from deploying existing therapies with far greater precision—anchored in mechanism-guided selection, fall-risk reduction, and sustained adherence, all integrated into a single patient-centered care model. A mechanistic understanding is the compass. Estrogen deficiency triggers three interlocking pathways—upregulated RANKL with suppressed OPG, heightened inflammatory cytokine signaling, and increased sclerostin expression—each mapping directly onto a therapeutic intervention (Figure 2). Bisphosphonates blunt osteoclast activity downstream; denosumab neutralizes RANKL at its source; romosozumab blocks sclerostin to unmask bone formation. This biological architecture underpins the risk-stratified treatment algorithm (Figure 4): moderate bone loss calls for antiresorptive suppression, whereas very high-risk patients with structural bone deficits require an anabolic-first strategy, followed by consolidation.

**Figure 5:**
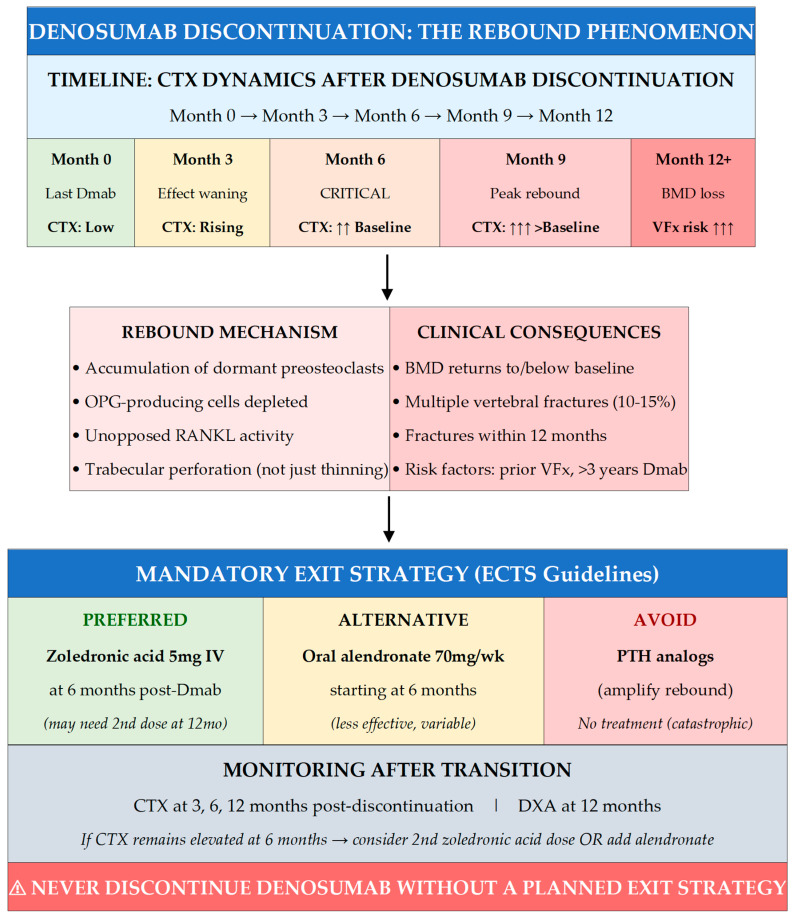
Denosumab Withdrawal: Rebound Mechanism and Transition Strategy with CTX Dynamics. This figure illustrates the timeline, mechanism, and management of the rebound phenomenon following denosumab discontinuation. Arrows indicate temporal progression of CTX dynamics (horizontal timeline) and the causal flow from rebound mechanism to clinical consequences to mandatory exit strategy (vertical arrows). Color coding: Dark blue header boxes indicate main topics (denosumab discontinuation, timeline, mandatory exit strategy, monitoring); Timeline boxes progress from green (Month 0, safe/low CTX) through yellow (Month 3, effect waning) to orange (Month 6, critical period) to coral/salmon (Month 9, peak rebound) to red (Month 12+, BMD loss and fracture risk); Yellow box explains the rebound mechanism (preosteoclast accumulation, OPG depletion, unopposed RANKL activity); Coral/red box shows clinical consequences (BMD loss, vertebral fractures); Light blue section presents the mandatory exit strategy with green (preferred: zoledronic acid), yellow (alternative: oral alendronate), and red (avoid: PTH analogs, no treatment) options; Gray box indicates monitoring protocol; Red warning banner emphasizes never discontinuing denosumab without a planned exit strategy. Abbreviations: BMD, bone mineral density; CTX, C-terminal telopeptide; Dmab, denosumab; ECTS, European Calcified Tissue Society; IV, intravenous; OPG, osteoprotegerin; PTH, parathyroid hormone; RANKL, receptor activator of nuclear factor-κB ligand; VFx, vertebral fracture. CTX dynamics and rebound mechanism from Bone et al. [76] and Cummings et al. [78]. Multiple vertebral fracture incidence (10–15%) from Cosman et al. [79] and Tsourdi et al. [80]. Exit strategy recommendations from ECTS guidelines, Tsourdi et al. [83].

Denosumab illustrates the value of mechanistic thinking. Its reversible inhibition of RANKL permits pre-osteoclast accumulation, explaining the rebound phenomenon and making an exit strategy mandatory (Figure 5).

Equally crucial—yet chronically undervalued—is fall prevention. More than 90% of hip fractures occur because of the fall, not because of uniquely fragile bone (Table 4). No anti-osteoporotic drug, however potent, can compensate for unaddressed fall risk. A care model that prioritizes pharmacology while ignoring biomechanics is inherently incomplete.

Finally, contextualization of MRONJ risk (Table 5) matters for practice: osteoporosis-level dosing carries a risk roughly 100-fold lower than oncology regimens. Fear of an exceedingly rare complication must never eclipse the far greater danger of untreated disease. The key practice points that follow distill this framework into pragmatic, evidence-based recommendations ready for clinical implementation—where mechanism meets bedside decision-making, and fracture prevention becomes genuinely holistic. Implementation of systematic screening programs, evidence-based treatment algorithms, proactive MRONJ counseling, planned denosumab exit strategies, and comprehensive fall prevention initiatives can substantially reduce the burden of osteoporotic fractures in postmenopausal women. The ultimate goal is to make the first fracture the last fracture—or better yet, to prevent the first fracture entirely [91].

## 9. Key Points

- Estrogen deficiency drives postmenopausal bone loss through three interconnected mechanisms: ↑RANKL/↓OPG ratio, pro-inflammatory cytokines, and ↑sclerostin—each representing a therapeutic target.
- Screen all women ≥65 years; screen younger postmenopausal women with risk factors (prior fracture, parental hip fracture, glucocorticoids, low BMI, smoking).
- Match treatment intensity to risk: HIGH risk → antiresorptive first; VERY HIGH risk (recent fracture, T-score ≤ −3.0) → anabolic first, then consolidate.
- MRONJ risk with osteoporosis-dose therapy is ~1:10,000–100,000/year—100× lower than oncology dosing; routine dental care can proceed; fear should not preclude treatment.
- Never stop denosumab without exit strategy: transition to zoledronic acid at 6 months to prevent rebound vertebral fractures (10–15% incidence without transition).

## 10. Key Practice Points

1. WHO SHOULD BE SCREENED

- All women aged ≥ 65 years (universal screening).
- Postmenopausal women <65 years with risk factors: prior fragility fracture, parental hip fracture, low body weight (<58 kg or BMI < 20), current smoking, excess alcohol (≥3 units/day), rheumatoid arthritis, early menopause (<45 years), or FRAX ≥ 10%.
- Any patient on glucocorticoids (≥5 mg/day for ≥ 3 months), aromatase inhibitors, or with conditions causing secondary osteoporosis.
- Patients with vertebral fractures or height loss (>4 cm historical or >2 cm prospective) on imaging.

2. SIMPLE TREATMENT ALGORITHM BY RISK CATEGORY

- HIGH RISK (T-score ≤ −2.5, prior fracture, FRAX above threshold): Start antiresorptive therapy—oral bisphosphonates as first-line (alendronate, risedronate); alternatives include IV zoledronic acid annually or denosumab every 6 months.
- VERY HIGH RISK (T-score ≤ −3.0, recent fracture < 24 months, multiple vertebral fractures, fracture on therapy): Start osteoanabolic therapy first—romosozumab × 12 months (if no cardiovascular disease) or teriparatide/abaloparatide × 24 months, THEN consolidate with antiresorptive.
- ALL PATIENTS: Ensure calcium 1000–1200 mg/day, vitamin D 800–2000 IU/day (target 25(OH)D ≥ 50 nmol/L), weight-bearing exercise, fall prevention, and smoking/alcohol cessation.

3. HOW TO MANAGE MRONJ FEARS

- Contextualize the risk: MRONJ incidence with osteoporosis-dose therapy is 1 in 10,000 to 1 in 100,000 patient-years—comparable to background ONJ risk and far lower than risks of aspirin GI bleeding (1 in 1000/year) or untreated osteoporosis (17% lifetime hip fracture risk).
- Distinguish osteoporosis dosing from oncology dosing: Denosumab 60 mg q6mo or zoledronic acid 5 mg/year (osteoporosis) carries 100-fold lower MRONJ risk than denosumab 120 mg monthly or zoledronic acid 4 mg monthly (cancer).
- Reassure patients and dentists: Routine dental care including extractions CAN proceed; good oral hygiene actually REDUCES MRONJ risk; drug holidays before dental procedures are generally NOT necessary for bisphosphonates.
- Emphasize the alternative: Refusing osteoporosis treatment due to MRONJ fear exposes patients to far greater, more certain harm from fractures—the benefit–risk ratio strongly favors treatment.

4. MANDATORY PLANNING FOR DENOSUMAB EXIT STRATEGIES

- NEVER discontinue denosumab without a planned exit strategy—rebound bone turnover causes 10–15% incidence of multiple vertebral fractures within 12 months of stopping.
- Preferred transition: Administer zoledronic acid 5 mg IV at 6 months after the last denosumab dose (when due for next injection); may require second dose at 12 months if CTX remains elevated.
- Monitor with CTX at 3, 6, and 12 months post-discontinuation; DXA at 12 months.
- AVOID switching to PTH analogs (amplify rebound); oral bisphosphonates are less effective than IV zoledronic acid for preventing rebound.
- Counsel patients BEFORE starting denosumab about this long-term commitment and transition requirements.

5. FALL PREVENTION IS FRACTURE PREVENTION

- Over 90% of hip fractures result from falls—address both bone fragility AND fall risk for comprehensive fracture prevention.
- Key interventions: Exercise programs with balance training (Tai Chi reduces falls by 40–50%), medication review (reduce sedatives, manage orthostatic hypotension), vision correction, home safety modifications, appropriate footwear.
- Screen with Timed Up and Go test (>12 s indicates increased risk) and fall history (≥2 falls or 1 injurious fall in past year warrants comprehensive assessment).

## Figures and Tables

**Table 1:** Comprehensive Clinical Evaluation for Suspected Osteoporosis.

HISTORY	
Fracture History	• Prior fragility fractures (location, age, mechanism) • Recent fractures (<24 months) indicating imminent risk • Childhood fractures or recurrent fractures • Fractures at atypical sites or with minimal trauma
Family History	• Parental hip fracture (strong FRAX risk factor) • Osteoporosis in first-degree relatives • Kyphosis or height loss in parents • Osteogenesis imperfecta or other genetic bone disorders
Menstrual/Reproductive History	• Age at menarche and menopause • Early menopause (<45 years) or surgical menopause • Prolonged amenorrhea (>12 months premenopausal) • History of eating disorders or female athlete triad • Hormone replacement therapy use (type, duration)
Medications	• Glucocorticoids (dose, duration; ≥5mg/day for ≥3 months) • Aromatase inhibitors (breast cancer treatment) • Anticonvulsants (phenytoin, phenobarbital, carbamazepine) • Proton pump inhibitors (long-term use) • Thyroid hormone (excess replacement) • GnRH agonists, medroxyprogesterone, heparin (long-term) • Prior osteoporosis treatment (agent, duration, response)
Lifestyle Factors	• Smoking status (current, former, pack-years) • Alcohol intake (≥3 units/day is FRAX risk factor) • Physical activity level and weight-bearing exercise • Calcium intake (dietary + supplements) • Vitamin D sources (sun exposure, diet, supplements) • Immobilization or sedentary lifestyle
Medical Conditions	• Rheumatoid arthritis (independent FRAX risk factor) • Other inflammatory conditions (SLE, IBD, ankylosing spondylitis) • Endocrine disorders (hyperthyroidism, hyperparathyroidism, Cushing) • Type 1 or Type 2 diabetes mellitus • Chronic kidney disease (stage, on dialysis?) • Malabsorption (celiac disease, gastric bypass, IBD) • Chronic liver disease • COPD and other chronic pulmonary disease • HIV infection • Malignancy (myeloma, breast/prostate cancer, bone metastases)
Fall Risk Assessment	• Falls in the past 12 months (number, circumstances) • Balance and gait problems • Visual impairment • Neurological conditions (stroke, Parkinson, neuropathy) • Medications affecting balance (sedatives, antihypertensives) • Home hazards and footwear
Dental History	• Current dental health status • Planned dental procedures (extractions, implants) • History of periodontal disease • Denture use and fit
PHYSICAL EXAMINATION	
Anthropometrics	• Current height (compare to historical maximum) • Height loss > 4 cm historical or >2 cm prospective → vertebral imaging • Weight and BMI (<20 kg/m^2^ is risk factor) • Recent unintentional weight loss
Spine Examination	• Thoracic kyphosis (dowager’s hump) • Rib-pelvis distance (<2 fingerbreadths suggests vertebral fracture) • Occiput-to-wall distance (>0 cm suggests kyphosis) • Spinal tenderness (may indicate acute fracture)
Musculoskeletal	• Muscle mass and strength (sarcopenia assessment) • Joint deformities suggesting inflammatory arthritis • Thigh or groin pain (may indicate atypical femoral fracture)
Balance and Mobility	• Timed Up and Go test (>12 s indicates fall risk) • Tandem stand and single leg stance • Gait assessment (speed, stability, use of assistive devices)
Signs of Secondary Causes	• Cushingoid features (moon facies, striae, buffalo hump) • Thyroid enlargement or signs of hyperthyroidism • Blue sclerae (osteogenesis imperfecta) • Signs of malabsorption or chronic disease
INVESTIGATIONS	
Baseline Laboratory (All Patients)	• Complete blood count • Serum calcium, phosphate, albumin • Alkaline phosphatase (bone-specific if available) • Creatinine and eGFR • 25-hydroxyvitamin D • TSH
Additional Tests (Based on Clinical Suspicion)	• PTH (if calcium abnormal or CKD) • Serum protein electrophoresis (if myeloma suspected) • 24 h urinary calcium (hypercalciuria, malabsorption) • Celiac serology (TTG-IgA) • Testosterone (men), FSH/LH (premenopausal women) • 24 h urinary free cortisol (if Cushing suspected) • Bone turnover markers (CTX, P1NP) for monitoring
Imaging	• DXA of lumbar spine, hip, and 33% radius • Vertebral fracture assessment (VFA) or lateral spine X-ray • Trabecular bone score (TBS) if available • Spine MRI if acute fracture or malignancy suspected
**Risk Assessment Tools**	• FRAX score (with and without BMD) • Apply FRAX adjustments if needed (TBS, glucocorticoid dose) • Compare to country-specific intervention thresholds

Abbreviations: BMD, bone mineral density; BMI, body mass index; CKD, chronic kidney disease; COPD, chronic obstructive pulmonary disease; CTX, C-terminal telopeptide; DXA, dual-energy X-ray absorptiometry; eGFR, estimated glomerular filtration rate; FSH, follicle-stimulating hormone; FRAX, Fracture Risk Assessment Tool; GnRH, gonadotropin-releasing hormone; IBD, inflammatory bowel disease; LH, luteinizing hormone; MRI, magnetic resonance imaging; P1NP, procollagen type 1 N-terminal propeptide; PTH, parathyroid hormone; SLE, systemic lupus erythematosus; TBS, trabecular bone score; TSH, thyroid-stimulating hormone; TTG, tissue transglutaminase; VFA, vertebral fracture assessment.

**Table 2:** Indications for DXA Bone Mineral Density Testing.

**Universal Screening Indications**
• All women aged ≥ 65 years • All men aged ≥ 70 years
**Postmenopausal Women <65 Years: Screen if ANY of the Following Present**
• Prior fragility fracture (after age 50) • Parental history of hip fracture • Low body weight (<58 kg) or BMI < 20 kg/m^2^ • Current smoking • Excess alcohol intake (≥3 units/day) • Rheumatoid arthritis or other inflammatory arthritis • Early menopause (<45 years) or prolonged premenopausal amenorrhea • FRAX 10-year major osteoporotic fracture probability ≥ 10% (without BMD)
**Medical Conditions and Medications Warranting DXA**
• Glucocorticoid therapy: ≥5 mg prednisone equivalent daily for ≥3 months (current or planned) • Aromatase inhibitor therapy for breast cancer • Androgen deprivation therapy (in men) • Primary hyperparathyroidism • Hyperthyroidism or excessive thyroid hormone replacement • Hypogonadism or premature ovarian insufficiency • Malabsorption syndromes (celiac disease, inflammatory bowel disease, gastric bypass) • Chronic kidney disease (CKD-MBD assessment) • Chronic liver disease • Organ transplantation • Type 1 diabetes mellitus • Anorexia nervosa or severe malnutrition • HIV infection (particularly with antiretroviral therapy)
**Radiographic or Clinical Findings Warranting DXA**
• Vertebral fracture or deformity on imaging (incidental or symptomatic) • Height loss > 4 cm (historical) or >2 cm (prospective) • Kyphosis suggesting vertebral fracture • Radiographic osteopenia on any imaging study
**Monitoring Indications**
• Monitoring response to osteoporosis treatment (typically every 1–2 years initially) • Monitoring bone loss during drug holiday • Detecting bone loss in untreated patients with osteopenia (every 2–5 years based on baseline)

Abbreviations: BMD, bone mineral density; BMI, body mass index; CKD-MBD, chronic kidney disease-mineral and bone disorder; DXA, dual-energy X-ray absorptiometry; FRAX, Fracture Risk Assessment Tool; HIV, human immunodeficiency virus.

**Table 3:** Comparison of Pharmacological Treatments for Postmenopausal Osteoporosis.

Agent	Mechanism	Dose/Route	Fracture Reduction	Advantages	Limitations	Special Notes
Alendronate	Antiresorptive (BP)	70 mg PO weekly	VF 44%, NVF 25%, HF 40%	Extensive data; Low cost; Generic	GI effects; Complex dosing	Avoid CrCl < 35; Drug holiday OK
Zoledronic Acid	Antiresorptive (BP)	5 mg IV yearly	VF 70%, NVF 25%, HF 41%	Annual; No GI; Best adherence	Acute phase rxn; IV access	Post-hip Fx proven; Longest holiday
Denosumab	Anti-RANKL	60 mg SC q6mo	VF 68%, NVF 20%, HF 40%	OK in CKD; Progressive gains	Rebound if stopped; Hypocalcemia	MUST transition to BP if stopping
Teriparatide	Anabolic (PTH)	20 μg SC daily × 24 mo	VF 65%, NVF 35%	Builds bone; Best for severe	Daily inj; Cost; 24 mo limit	MUST follow with AR; CV safe
Romosozumab	Anti-sclerostin	210 mg SC monthly × 12 mo	VF 73%, CF 36%	Fastest BMD gain; Dual effect	CV signal; 12 mo limit; Cost	CI if prior MI/stroke; MUST follow AR

Abbreviations: AR, antiresorptive; BP, bisphosphonate; CF, clinical fracture; CI, contraindicated; CKD, chronic kidney disease; CrCl, creatinine clearance; CV, cardiovascular; Fx, fracture; GI, gastrointestinal; HF, hip fracture; IV, intravenous; MI, myocardial infarction; NVF, non-vertebral fracture; PO, oral; PTH, parathyroid hormone; rxn, reaction; SC, subcutaneous; VF, vertebral fracture. Fracture risk reduction data from pivotal trials as summarized in Eastell et al. [20] and Compston et al. [2]. BMD gains from respective phase 3 trials.

**Table 4:** Evidence-Based Fall Prevention Interventions.

Intervention	Key Components	Expected Benefit
**Exercise Programs**	Balance training, strength training, Tai Chi, gait training; supervised initially; sustained participation	**↓ Falls 20** **–** **40%; Tai Chi ↓ 40** **–** **50%**
**Medication Review**	Reduce polypharmacy; minimize sedatives, hypnotics, antihypertensives causing orthostasis; time diuretics appropriately	**↓ Falls 20** **–** **30% with psychotropic withdrawal**
**Vision Correction**	Annual eye exam; cataract surgery when indicated; single-vision glasses for mobility; optimize home lighting	**↓ Falls with cataract surgery; single-vision glasses ↓ outdoor falls**
**Home Safety Modification**	Remove loose rugs; install grab bars, handrails; improve lighting; clear pathways; non-slip mats in bathrooms	**↓ Falls 20** **–** **30% (especially with OT assessment)**
**Footwear Optimization**	Low heels (<2.5 cm); firm heel counter; slip-resistant soles; secure fastening; avoid slippers, bare feet	**Reduces fall risk; limited direct trial evidence**
**Orthostatic Hypotension Management**	Slow positional changes; adequate hydration; compression stockings; medication adjustment; fludrocortisone if refractory	**Reduces syncope and falls; individual response variable**
**Vitamin D Supplementation**	700–1000 IU daily; target 25(OH)D > 60 nmol/L; avoid annual bolus dosing	**↓ Falls 14** **–** **20% (greater if deficient at baseline)**
**Hip Protectors**	Padded undergarments deflecting impact from greater trochanter; best evidence in nursing home residents	**↓ Hip fractures in institutions if worn; poor adherence limits effectiveness**
**Multifactorial Assessment**	Comprehensive geriatric assessment addressing multiple risk factors simultaneously; individualized intervention plan	**↓ Falls 20** **–** **30%; most effective approach for high-risk individuals**

Abbreviations: OT, occupational therapy; 25(OH)D, 25-hydroxyvitamin D. Green shading indicates interventions with strong evidence; yellow indicates limited or conditional evidence. Exercise data from Sherrington et al. [44]; medication review from Woolcott et al. [42]; vision from Harwood et al. [43]; home safety from Gillespie et al. [45]; vitamin D from Bischoff-Ferrari et al. [48]; multifactorial assessment from Gillespie et al. [45].

**Table 5:** Comparative Risk Perspective: MRONJ in Context.

Clinical Scenario	Approximate Risk
***MRONJ Risk by Treatment Setting***	
Oral bisphosphonates for osteoporosis	**1 in 10,000 to 1 in 100,000 per year**
IV zoledronic acid 5 mg yearly (osteoporosis)	**1 in 10,000 to 1 in 50,000 per year**
Denosumab 60 mg q6mo (osteoporosis)	**1 in 10,000 to 1 in 100,000 per year**
IV zoledronic acid 4 mg monthly (cancer)	**1–2% (1 in 50 to 1 in 100)**
Denosumab 120 mg monthly (cancer)	**1–5% (1 in 20 to 1 in 100)**
***Comparative Common Medical Risks***	
GI bleeding from low-dose aspirin	1 in 1000 per year
VTE from oral contraceptives	1 in 1000 per year
Background ONJ (no bone medications)	~1 in 100,000 per year
***Risks of NOT Treating Osteoporosis***	
Lifetime hip fracture risk (women age 50)	**1 in 6 (17%)**
One-year mortality after hip fracture	**20–25%**
Lifetime any osteoporotic fracture risk (women)	**1 in 2 (50%)**

Abbreviations: GI, gastrointestinal; IV, intravenous; ONJ, osteonecrosis of the jaw; VTE, venous thromboembolism. Green shading indicates very low risk; orange indicates elevated risk; red indicates high risk of adverse outcome. MRONJ incidence data from Khan et al. [32], Ruggiero et al. [33], and Fassio et al. [64]. Comparative medical risks from standard epidemiological sources. Osteoporosis fracture risk data from Johnell & Kanis [5] and Compston et al. [2].

## Data Availability

No new data were created or analyzed in this study.
